# Non-melancholic depressive symptoms are associated with above average fat mass index in the Helsinki birth cohort study

**DOI:** 10.1038/s41598-022-10592-3

**Published:** 2022-04-28

**Authors:** Mia D. Eriksson, Johan G. Eriksson, Päivi Korhonen, Minna K. Salonen, Tuija M. Mikkola, Eero Kajantie, Niko S. Wasenius, Mikaela von Bonsdorff, Hannu Kautiainen, Merja K. Laine

**Affiliations:** 1 Primary Health Care Unit, Helsinki University Hospital (HUS), Helsinki, Finland; 2 Folkhälsan Research Center, Helsinki, Finland; 3 Department of General Practice and Primary Health Care, University of Helsinki and Helsinki University Hospital, Helsinki, Finland; 4 Department of Obstetrics and Gynecology and Human Potential Translational Research Programme, Yong Loo Lin School of Medicine, National University of Singapore, Singapore, Singapore; 5 Singapore Institute for Clinical Sciences (SICS), Agency for Science, Technology and Research (A*STAR), Singapore, Singapore; 6 Department of General Practice, Turku University Hospital and University of Turku, Turku, Finland; 7 Department of Public Health Solutions, Public Health Promotion Unit, Finnish Institute for Health and Welfare, Helsinki, Finland; 8 Clinicum, Faculty of Medicine, University of Helsinki, Helsinki, Finland; 9 PEDEGO Research Unit, MRC Oulu, Oulu University Hospital and University of Oulu, Oulu, Finland; 10 Department of Clinical and Molecular Medicine, Norwegian University of Science and Technology, Trondheim, Norway; 11 Gerontology Research Center and Faculty of Sport and Health Sciences, University of Jyväskylä, Jyväskylä, Finland; 12 Institute of Public Health and Clinical Nutrition, University of Eastern Finland, Kuopio, Finland

**Keywords:** Medical research, Signs and symptoms, Risk factors, Psychology, Metabolism, Neurophysiology

## Abstract

There is an existing link between two of the most common diseases, obesity and depression. These are both of great public health concern, but little is known about the relationships between the subtypes of these conditions. We hypothesized that non-melancholic depressive symptoms have a stronger relationship with both body composition (lean mass and fat mass) and dysfunctional glucose metabolism than melancholic depression. For this cross-sectional study 1510 participants from the Helsinki Birth Cohort Study had their body composition evaluated as lean mass and fat mass (Lean Mass Index [LMI, kg/m^2^] + Fat Mass Index [FMI kg/m^2^] = Body Mass Index). Participants were evaluated for depressive symptoms utilizing the Beck depression inventory, and had laboratory assessments including an oral glucose tolerance test. Higher than average FMI was associated with a higher percentage (mean [%], 95% CI) of participants scoring in the depressive range of the Beck depression inventory (20.2, 17.2–23.2) compared to those with low FMI (16.3, 13.8–18.9; *p* = 0.048) when adjusted for age, sex, education, and fasting plasma glucose concentration. Higher FMI was associated with a higher likelihood of having depressive symptoms (OR per 1-SD FMI = 1.37, 95% CI 1.13–1.65), whereas higher LMI was associated with a lower likelihood of having depressive symptoms (OR per 1-SD LMI = 0.76, 95% CI 0.64–0.91). Participants with an above average FMI more frequently (mean [%], 95% CI) had non-melancholic depressive symptoms (14.7, 11.8–17.7) as compared to those with low FMI (9.7, 7.6–11.9; *p* = 0.008) regardless of LMI levels. There was no difference between the body composition groups in the likelihood of having melancholic depressive symptoms. The non-melancholic group had higher (mean [kg/m^2^], SD) FMI (9.6, 4.1) than either of the other groups (BDI < 10: 7.7, 3.1; melancholic: 7.9, 3.6; *p* < 0.001), and a higher (mean [mmol/l], SD) 2-h glucose concentration (7.21, 1.65) than the non-depressed group (6.71, 1.70; *p* = 0.005). As hypothesized, non-melancholic depressive symptoms are most closely related to high fat mass index and dysfunctional glucose metabolism.

## Introduction

Obesity and depression are both among the most common diseases globally^1^, and they have been known to frequently co-occur implying a close relationship between the two^2^. Overweight, obesity and depression are of significant public health concern^1,3,4^ as their prevalence continues to increase^1,3^ and further contribute to the overall economic burden on society^1,3,4^.

High body mass index (BMI) both accounts for a large portion of disability-adjusted life years compared to other risk factors, and has an increasing summary exposure, which is of concern for current and future health^5^. There were high positive rates of change for high BMI globally from 2010 to 2019^5^. In 2019 high BMI accounted for more than 6 disability-adjusted life years for each sex, and was highest among the middle and high socio-demographics. High BMI also accounted for around 5 million global attributable deaths for men and women together in 2019^5^.

By the year 2030 depression has been predicted to be the leading cause of burden of disease^6^. Currently, on a global level, depression affects more than 322 million individuals^7^. It contributes enormously to the overall burden of disease^8^, and is the current worldwide leading cause of years being lost to disability^7^. Depression also comes with a two-fold increased risk of death that previous studies have not been able to explain to be a result of either behavior or physical illness^9^.

Depression is a heterogenous condition that can be divided into subtypes; melancholic and non-melancholic depression^10^. According to the DSM-IV melancholic depression is characterized by a lack of interest in usual pleasure without improvement when good things happen^11^. Furthermore, the depression is frequently worse in the morning, includes early awakening, psychomotor symptoms, weight loss, and excessive guilt^11^. Non-melancholic depression, on the other hand, more frequently presents with mood reactivity and sensitivity to rejection, along with leaden paralysis, increased sleep and weight gain^11^. These subtypes are known to differ in their pathophysiology^10,12^. Metabolic and inflammatory dysregulations have been implicated in the case of non-melancholic depression, with hypothalamic–pituitary–adrenal (HPA)-axis dysregulations being linked to melancholic depression^10,12^. Others suggest that the HPA-axis may be linked to both, where overactivity is related to melancholic depression and underactivity is related to non-melancholic depression^2^.

The existence of a relationship between body composition and depressive symptoms has been well established^2–4,12–18^. One hypothesis is that a relationship between obesity and depression may exist due to central and peripheral inflammation^2^. Relationships between depressive symptoms and higher fat mass, or lower muscle mass have been reported^13^. By focusing on depressive subtypes and studying how they differ in regard to body composition we may be able to learn more about the heterogeneity of depression.

There is an established relationship between depression and diabetes mellitus^19,20^ although this association may not extend to people with impaired glucose metabolism or undiagnosed diabetes^21^. Some studies suggest that the association with impaired glucose metabolism is stronger in depressed individuals with anhedonia than in those without^22^. There is also some indication that specifically non-melancholic depression is associated with dysfunctional glucose metabolism^12,23^. Impaired glucose metabolism is also well known to be associated with obesity, metabolic syndrome, and body composition^24,25^.

Previous studies have primarily focused on either the subtypes of depression or individual components of body composition, without taking both into consideration simultaneously. Existing research has shown that there is a difference in body composition and glucose metabolism between those with melancholic and non-melancholic depression^12^.

The aim of this study is to evaluate the relationships between body composition, expressed as lean mass and fat mass, and subtypes of depressive symptoms. Furthermore, we want to evaluate how glucose metabolism varies with non-melancholic and melancholic depressive symptoms. We hypothesize that non-melancholic depressive symptoms have a stronger relationship with both body composition and dysfunctional glucose metabolism than melancholic depressive symptoms.

## Materials and methods

### Participants

Helsinki Birth Cohort Study is composed of men and women born at either Helsinki University Hospital or Helsinki City Maternity Hospital in Helsinki, Finland between 1934 and 1944. The cohort consists of a total of 13,345 participants that attended child welfare clinics in Helsinki, with most participants also having attended school in Helsinki. More detailed information regarding birth, child welfare, and school records have been published elsewhere^26,27^. After receiving unique identification numbers by the Finnish government in 1971, 8760 individuals (4630 men and 4130 women) from this cohort, born at Helsinki University Hospital, were identified. In 2001–2004, 2902 subjects were selected for further studies using random-number tables. These participants were selected from the pool of the original cohort that were alive and still living in Finland. Of the subjects that were identified and selected, 2003 individuals participated in the study. Individuals with diabetes mellitus were excluded from the analyses due to the study design and research question. After exclusion of missing data and participants with high-sensitivity C-reactive protein (hsCRP) > 10 mg/l^28^, the final number of participants was 1510.

### Depressive symptoms

To assess depressive symptoms in the participants, the Beck Depression Inventory (BDI) was used. The BDI is a self-reported questionnaire consisting of 21 questions specific to depression^29^. The questionnaire is scored on a 0–63 point scale that has been validated to screen for mild to severe clinical depression with a ≥ 10 point cut-off^30^.

Those participants scoring ≥ 10 were classified as having either non-melancholic or melancholic depressive symptoms based on the presence or absence of melancholic symptoms according to the DSM-IV. We used the symptoms of sadness, past failure, loss of pleasure, guilty feelings, punishment feelings, loss of interest, irritability, changes in sleep and appetite to assess melancholy in accordance with prior publications^23,31,32^. Participants were classified as either having non-melancholic or melancholic depressive symptoms based on which summary score for the symptoms was greater^23,31,32^.

### Anthropometry and body composition

Height and weight were obtained using a Kawi stadiometer and a Seca Alpha 770 scale, respectively. Measurements were obtained with participants wearing light indoor clothing, and no shoes. The height and weight were measured to the nearest 0.1 cm and 0.1 kg, respectively. BMI (kg/m^2^) was calculated as weight (kg), divided by height squared (m^2^). Each participant had their body composition assessed by an eight-polar tactile electrode bio-impedance system (InBody 3.0, Biospace Co, Ltd, Seoul, Korea). Fat mass index (FMI, kg/m^2^) was calculated as measured fat mass (kg), divided by height squared (m^2^). Lean mass index (LMI, kg/m^2^) was calculated as weight minus fat mass (kg), divided by height squared (m^2^). FMI + LMI = BMI. FMI and LMI had their z-scores stratified by sex and then combined once standardized, creating four distinct categories for body composition; (A) above mean FMI and below mean LMI, (B) above mean FMI and above mean LMI, (C) below mean FMI and below mean LMI, (D) below mean FMI and above mean LMI^33^.

### Other measurements

Participants had blood drawn after an overnight fast for laboratory assessment. These included hsCRP, glucose, insulin, and lipids. hsCRP was measured using a photometric immunochemical method. Plasma glucose was measured by a hexokinase method at 0 min (fasting), 30 min and 120 min after a 75 g oral glucose tolerance test (OGTT). Fasting plasma insulin was measured by two-site immunometric assay^34^. Total cholesterol and lipids were measured by standard enzymatic methods^35,36^. Homeostatic Model Assessment for Insulin Resistance (HOMA-IR) was calculated as [(fasting plasma glucose in mmol/l × fasting plasma insulin in mU/l)/22.5]^37,38^.

Blood pressure was measured with the subject in a seated position. Two measurements were obtained from the right arm with a standard sphygmomanometer. The reported values are the means of the two readings. Pulse pressure was calculated as the difference between the mean systolic pressure and mean diastolic pressure.

Physical activity was assessed using the validated 12‐month leisure‐time physical activity (LTPA) questionnaire from the Kuopio Ischaemic Heart Disease Risk Factor Study^39,40^. Participants reported their activity over the past 12 months as typical intensity, frequency (occasions per month), and average duration. Utilizing available databases, values for the metabolic equivalent of task (MET; 1 MET = 3.5 ml O_2_/kg/min) were assigned to each activity^41^. Total LTPA in MET-hours per week were calculated by multiplying MET by average duration and frequency divided by the weeks.

Information regarding alcohol consumption and smoking, in addition to socioeconomic factors (years of education, and cohabitation) and health status were obtained through questionnaires.

### Ethics

The Coordinating Ethical Committee of the Hospital District of Helsinki and Uusimaa has approved the study protocol (344/E3/2000), and all study procedures followed the ethical guidelines of the declaration of Helsinki. Written informed consent was obtained from each subject prior to their participation.

### Statistical analysis

The descriptive statistics were presented as means with SDs or as counts with percentages. Statistical comparisons between groups were done using analysis of variance (ANOVA), and a chi-square test. Results were also analyzed using factorial (two between-subjects factors: FMI and LMI) ANOVA and logistic models. Models included main effects of FMI and LMI and their interaction. When adjusted models were used, analysis of covariance was applied (age, sex, education, and fasting plasma glucose concentration as covariates). Hommel’s adjustment was applied where appropriate to correct levels of significance for multiple testing (post hoc). Hommel's adjustment was used because it is more powerful than alternative procedures, including the Bonferroni, Holm's and Hochberg's procedures^42^ Relationship between BDI (≥ 10) and body composition was modeled using adjusted logistic models. The bootstrap method was used when the theoretical distribution of the test statistics was unknown, or in the case of violation of the assumptions (e.g. non-normality). Correlation coefficients with 95% CI were calculated by using the Pearson method. The normality of variables was evaluated graphically and by using the Shapiro–Wilk W test. Stata 17 (StataCorp LP; College Station, Texas, USA) statistical package was used for the analysis.

## Results

We analyzed and compared demographic as well as lifestyle related variables of subjects excluded from the study (n = 182) and compared them to the study participants (n = 1510) (Appendix 1). There was a significant difference in age and educational attainment between the groups, however this difference was minimal and not of clinical relevance.

**Figure 1:**
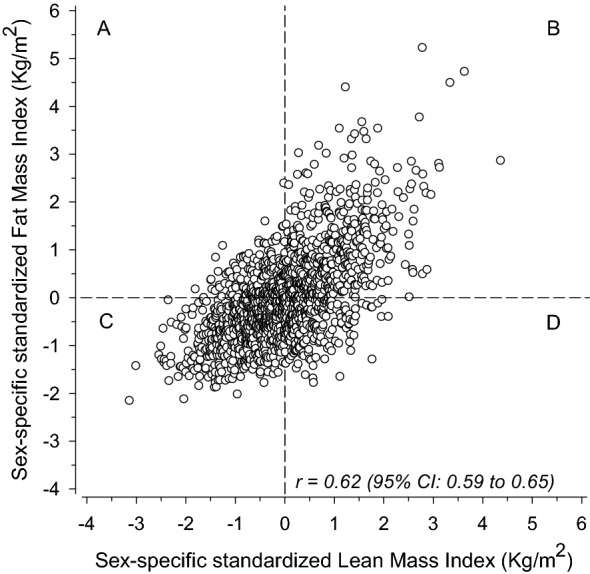
Categorizing participants into groups based on sex-specific standardized Fat Mass Index (FMI) and Lean Mass Index (LMI). The dashed lines represent mean values. Both axes represent z-scores. The letters A, B, C, D represent the 4 body composition groups. A: above mean fat mass index (FMI) and below mean lean mass index (LMI), B: above mean FMI and above mean LMI, C: below mean FMI and below mean LMI, D: below mean FMI and above mean LMI.

Figure 1 shows the sex-specific standardized distribution of all participants in relation to FMI and LMI. The correlation coefficient between FMI and LMI was 0.62 (95% CI 0.59–0.65). Groups A (high FMI and low LMI), B (high FMI and high LMI), C (low FMI and low LMI), and D (low FMI and high LMI) included 197, 470, 580, and 263 cohort members, respectively.

Table 1 shows the cohort characteristics according to body composition groups. An interaction between FMI and LMI was found for sex (*p* < 0.001). The proportion of women was higher in groups B and C (high FMI–high LMI, and low FMI–low LMI) than in groups D and A (low LMI–high FMI, and high LMI–low FMI). Those with high FMI had higher blood pressure, heart rate, triglyceride concentration, hsCRP, and BDI score, but lower HDL-cholesterol concentration. Those with high LMI also had higher blood pressure and triglyceride concentration, along with lower HDL-cholesterol concentration and heart rate. However, no differences in hsCRP and BDI were observed. There was an effect for high FMI on glucose concentrations at 0, 30, and 120 min after an OGTT, along with fasting insulin concentration and HOMA-IR. High LMI was related to higher fasting glucose, fasting insulin, and HOMA-IR, but there was no relation to post-load glucose concentrations.

**Table 1:** Characteristics of participants by group.

	High FMI		Low FMI		*p* value		
	A	B	C	D	Main effect		Interaction
	Low LMI n = 197	High LMI n = 470	Low LMI n = 580	High LMI n = 263	FMI	LMI	
Women, n (%)	96 (49)	281 (60)	341 (59)	124 (47)	0.65	0.92	< 0.001
Age (years), mean (SD)	61.3 (3.2)	61.2 (3.0)	60.9 (2.9)	60.6 (2.5)	0.002	0.15	0.65
Education (years), mean (SD)	11.9 (3.6)	11.9 (3.4)	12.7 (3.8)	13.1 (3.7)	< 0.001	0.38	0.33
Cohabitating, n (%)	149 (76)	358 (76)	428 (74)	207 (79)	0.86	0.26	0.36
Current smoker, n (%)	42 (21)	103 (22)	136 (23)	75 (29)	0.075	0.26	0.39
Alcohol consumption, n (%)					0.023	0.005	0.12
0	18 (9)	32 (7)	45 (8)	7 (3)			
1–2/month	76 (39)	205 (44)	213 (37)	105 (40)			
≥ 1/week	102 (52)	229 (49)	321 (55)	150 (57)			
LTPA (METh/week), mean (SD)	37.8 (29.0)	35.7 (26.0)	40.1 (27.3)	40.8 (28.1)	0.016	0.65	0.37
BMI (kg/m^2^), mean (SD)	27.4 (1.4)	31.7 (3.5)	23.4 (1.9)	26.3 (1.5)	< 0.001	< 0.001	< 0.001
BP (mmHg), mean (SD)							
Systolic	146 (20)	148 (19)	139 (20)	143 (19)	< 0.001	0.007	0.42
Diastolic	90 (10)	92 (10)	86 (10)	88 (10)	< 0.001	0.002	0.60
Pulse pressure	56 (17)	57 (15)	53 (15)	55 (14)	0.016	0.16	0.49
Pulse (bpm)	70 (12)	69 (11)	69 (11)	67 (10)	0.008	0.003	0.16
Glucose (mmol/l), mean (SD)							
0 min	5.51 (0.57)	5.63 (0.56)	5.37 (0.54)	5.48 (0.53)	< 0.001	< 0.001	0.93
30 min	9.17 (1.69)	9.27 (1.52)	8.80 (1.73)	8.81 (1.60)	< 0.001	0.57	0.62
120 min	7.22 (1.67)	7.25 (1.65)	6.50 (1.65)	6.59 (1.75)	< 0.001	0.53	0.78
Fasting insulin (μU/ml) mean (SD)	11.1 (14.8)	13.8 (16.5)	7.2 (4.2)	7.8 (4.1)	< 0.001	0.008	0.10
HOMA-IR, mean (SD)	2.74 (3.51)	3.52 (4.48)	1.73 (1.08)	1.92 (1.07)	< 0.001	0.003	0.073
Total Cholesterol (mmol/l), mean (SD)	6.06 (1.11)	6.04 (1.07)	5.93 (0.97)	6.00 (1.03)	0.15	0.59	0.44
LDL-cholesterol (mmol/l), mean (SD)	3.78 (0.91)	3.78 (0.89)	3.61 (0.82)	3.77 (0.87)	0.083	0.10	0.10
HDL-cholesterol (mmol/l), mean (SD)	1.59 (0.42)	1.53 (0.38)	1.76 (0.44)	1.63 (0.42)	< 0.001	< 0.001	0.15
Triglycerides, (mmol/l), mean (SD)	1.51 (0.71)	1.66 (0.85)	1.23 (0.61)	1.33 (0.64)	< 0.001	0.001	0.53
hsCRP (mg/l), mean (SD)	2.94 (2.41)	2.79 (2.30)	1.59 (1.74)	1.78 (1.86)	< 0.001	0.85	0.15
Diseases, n (%)							
CVD	20 (10)	40 (9)	29 (5)	7 (3)	< 0.001	0.10	0.37
Pulmonary	18 (9)	59 (13)	48 (8)	24 (9)	0.23	0.23	0.52
RA	2 (1)	7 (1)	11 (2)	6 (2)	0.26	0.55	0.83
BDI, mean (SD)	6.0 (5.3)	6.2 (5.6)	5.4 (5.0)	4.9 (4.5)	< 0.001	0.54	0.20
BDI subtype, n (%)					0.44	0.19	0.18
< 10	159 (81)	367 (78)	480 (83)	230 (87)			
NMeD	28 (14)	76 (16)	64 (11)	19 (7)			
MeD	10 (5)	27 (6)	36 (6)	14 (5)			

Disease subgroups: *CVD*: angina pectoris, myocardial infarction, stroke; *Pulmonary*: asthma, chronic obstructive pulmonary disease.

*BDI* beck depression inventory, *BMI* body mass index, *BP* blood pressure, *bpm* beats per minute, *CVD* cardiovascular disease, *FMI* fat mass index, *HDL* high density lipoprotein, *HOMA-IR* homeostatic model assessment for insulin resistance, *hsCRP* high sensitivity C-reactive protein, *LDL* low density lipoprotein, *LMI* lean mass index, *LTPA* leisure-time physical activity, *MeD* melancholic depressive symptoms, *NMeD* non-melancholic depressive symptoms, *RA* rheumatoid arthritis, *SD* standard deviation.

Glucose measurements were taken after a 75 g oral glucose tolerance test.

Table 2 shows the characteristics of the participants according to which depressive subgroup they were classified into. The non-melancholic group had proportionally more women, and higher FMI (mean 9.6 kg/m^2^, 4.1 SD) than either of the other groups (BDI < 10: mean 7.7 kg/m^2^, 3.1 SD; melancholic: mean 7.9 kg/m^2^, 3.6 SD; *p* < 0.001). Compared to the non-depressed group they also had lower LMI, higher 2-h glucose concentration (non-melancholic: mean 7.21 mmol/l, 1.65; BDI < 10: mean 6.71 mmol/l, 1.70 SD; *p* = 0.005), and higher hsCRP. The melancholic group had lower total cholesterol, LDL-cholesterol, and blood pressure than either of the other two groups.

**Table 2:** Characteristics according to depressive subtypes.

	BDI < 10 [X]	NMeD [Y]	MeD [Z]	*p* value [multiple comparison]*
	N = 1236	N = 187	N = 87	
Women, n (%)	651 (53)	143 (76)	48 (55)	< 0.001 [X/Y, Y/Z]
Age (years), mean (SD)	61 (3)	62 (3)	61 (3)	0.061
Education (years), mean (SD)	12.5 (3.7)	11.7 (3.3)	11.9 (3.5)	0.005 [X/Y]
Cohabitating, n (%)	954 (77)	127 (68)	61 (70)	0.011 [X/Y]
Current smoker, n (%)	284 (23)	48 (26)	24 (28)	0.48
Alcohol consumption, n (%)				0.005 [X/Y, X/Z]
0	67 (5)	22 (12)	13 (15)	
1–2/month	489 (40)	76 (41)	34 (39)	
≥ 1/week	674 (55)	88 (47)	40 (46)	
LTPA (METh/week), mean (SD)	38 (27)	39 (32)	41 (28)	0.70
BMI (kg/m^2^), mean (SD)	26.9 (3.9)	28.2 (5.4)	26.8 (4.6)	< 0.001 [X/Y, Y/Z]
FMI, crude mean (SD)	7.7 (3.1)	9.6 (4.1)	7.9 (3.6)	< 0.001 [X/Y, Y/Z]
LMI, crude mean (SD)	19.1 (2.1)	18.6 (2.2)	18.8 (2.2)	0.006 [X/Y]
Blood pressure (mmHg), mean (SD)				
Systolic	144 (20)	146 (19)	137 (20)	0.004 [X/Z, Y/Z]
Diastolic	89 (10)	89 (10)	85 (9)	< 0.001 [X/Z, Y/Z]
Pulse pressure	55 (15)	56 (15)	53 (17)	0.20
Pulse (bpm)	69 (11)	70 (12)	69 (13)	0.67
Glucose (mmol/l), mean (SD)				
0 min	5.49 (0.54)	5.49 (0.61)	5.50 (0.62)	0.96
30 min	8.95 (1.62)	9.22 (1.69)	9.13 (1.98)	0.10
120 min	6.78 (1.70)	7.21 (1.65)	6.94 (1.72)	0.005 [X/Y]
Fasting insulin (μU/ml), mean (SD)	9.8 (11.8)	11.2 (11.3)	8.4 (5.1)	0.15
HOMA-IR, (mean (SD)	2.43 (3.12)	2.80 (2.86)	2.10 (1.34)	0.16
Total Cholesterol (mmol/l), mean (SD)	6.01 (1.03)	6.02 (1.05)	5.65 (0.99)	0.005 [X/Z, Y/Z]
LDL-cholesterol (mmol/l), mean (SD)	3.75 (0.86)	3.65 (0.88)	3.38 (0.79)	< 0.001 [X/Z, Y/Z]
HDL-cholesterol (mmol/l), mean (SD)	1.64 (0.42)	1.70 (0.43)	1.62 (0.49)	0.14
Triglycerides, (mmol/l), mean (SD)	1.40 (0.71)	1.50 (0.84)	1.45 (0.78)	0.24
hsCRP (mg/l), mean (SD)	2.11 (2.08)	2.49 (2.25)	2.29 (2.39)	0.045 [X/Y]
Diseases, n (%)				
CVD	61 (5)	20 (11)	15 (17)	< 0.001 [X/Y, X/Z]
Pulmonary	104 (8)	33 (18)	12 (14)	< 0.001 [X/Y]
RA	20 (2)	4 (2)	2 (2)	0.66
BDI, mean (SD)	3.7 (2.7)	14.4 (5.2)	13.8 (4.6)	

Disease subgroups: *CVD*: angina pectoris, myocardial infarction, stroke; *Pulmonary*: asthma, chronic obstructive pulmonary disease.

*BDI* beck depression inventory, *BMI* body mass index, *bpm* beats per minute, *CVD* cardiovascular disease, *FMI* fat mass index, *HDL* high density lipoprotein, *HOMA-IR* homeostatic model assessment for insulin resistance, *hsCRP* high sensitivity C-reactive protein, *LDL* low density lipoprotein, *LMI* lean mass index, *LTPA* leisure-time physical activity, *MeD* melancholic depressive symptoms, *NMeD* non-melancholic depressive symptoms, *RA* rheumatoid arthritis, *SD* standard deviation.

*Hommel’s multiple comparison procedure was used to correct significance levels for post hoc testing (*p* < 0.05).

**Figure 2:**
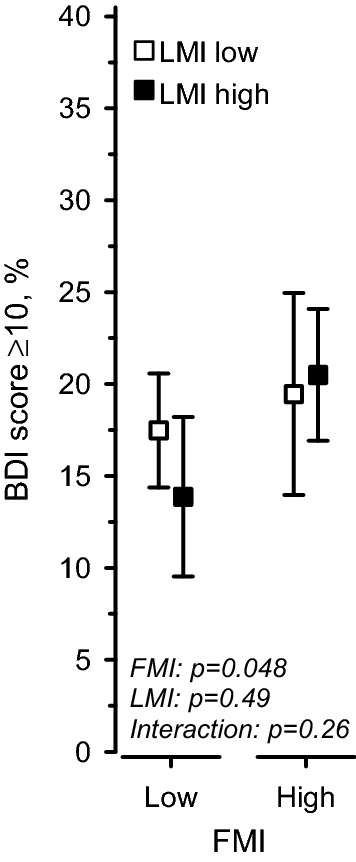
Percentage of BDI ≥ 10 according to body composition group. *FMI* fat mass index, *LMI* lean mass index, *BDI* beck depression inventory. The model has been adjusted for age, sex, education, and fasting plasma glucose concentration.

Figure 2 shows the percentage of participants in each of the four groups (A, B, C, D) that scored ≥ 10 on the BDI. An above average FMI was associated with a higher percentage (mean [%], 95% CI) of participants scoring in the depressive range (20.2, 17.2–23.2) compared to those with low FMI (16.3, 13.8–18.9; *p* = 0.048) when adjusted for age, sex, education, and fasting plasma glucose concentration. No effect was seen by LMI (*p* = 0.49). No interaction was found (*p* = 0.26). The analyses were adjusted for age, sex, education, and fasting plasma glucose concentration.

**Figure 3:**
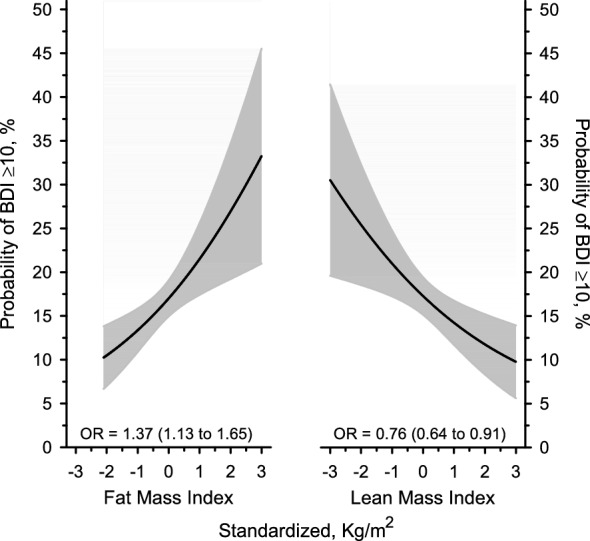
Relationship between BDI ≥ 10 according to body composition. *BDI* beck depression inventory, *OR* odds ratio. Odds ratio is presented per 1-SD. Models have been adjusted for age, sex, education, and fasting plasma glucose concentration.

Figure 3 shows the probability of scoring ≥ 10 on the BDI as a continuous function of standardized FMI and LMI adjusted for age, sex, education, and fasting plasma glucose concentration. Higher FMI was associated with a higher likelihood of having depressive symptoms (OR per 1 SD FMI = 1.37 95% CI 1.13–1.65). Higher LMI was associated with lower likelihood of having depressive symptoms (OR per 1 SD LMI = 0.76 95% CI 0.64–0.91).

**Figure 4:**
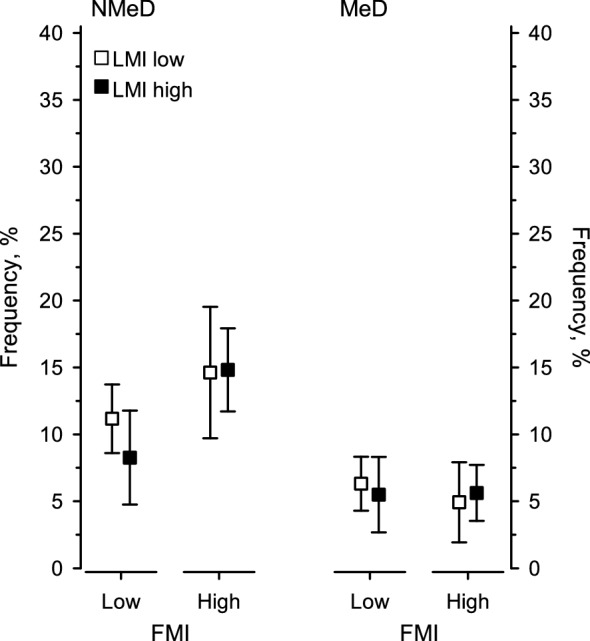
Frequency of depressive subtypes in relation to body composition as represented by mean and standard deviation. *BDI* beck depression inventory, *FMI* fat mass index, *LMI* lean mass index, *MeD* melancholic depressive symptoms, *NMeD* non-melancholic depressive symptoms. Models have been adjusted for age, sex, education, and fasting plasma glucose concentration. NMeD: FMI *p* = 0.008, LMI *p* = 0.38, interaction *p* = 0.31. MeD: FMI *p* = 0.83, LMI *p* = 0.93, interaction *p* = 0.52.

Figure 4 has been adjusted for age, sex, education, and fasting plasma glucose concentration. Participants with a high FMI more often (mean [%], 95% CI) had non-melancholic depressive symptoms (14.7, 11.8–17.7) compared to those with low FMI (9.7, 7.6–11.9; *p* = 0.008) regardless of LMI levels (*p* = 0.38). No interaction was found (*p* = 0.31). No differences were found in the frequency of having melancholic depressive symptoms between the body composition groups (FMI *p* = 0.83, LMI *p* = 0.93). No interaction was found (*p* = 0.52).

## Discussion

As one of the two subcomponents of BMI, FMI showed a distinct relationship with a higher frequency of participants scoring in the depressive range on the BDI. When analyzing the subtypes of depressive symptoms separately no such relationship was found in the melancholic group. In accordance with our hypothesis, we found that the group with non-melancholic depressive symptoms showed a more pronounced relationship with body composition than those with melancholic depressive symptoms. More specifically, non-melancholic depressive symptoms were associated with higher FMI. This may suggest that the combined effect showing a relationship between high FMI and depressive symptoms was explained by the findings in the non-melancholic group. This is an important finding, as prior research has established the relationship between increased fat mass and depression^13^. Our findings suggest that this may be limited to non-melancholic depressive symptoms rather than overall depressive symptoms or melancholic depressive symptoms.

Furthermore, our findings suggest that body fat mass and lean mass have opposite relationships with the prevalence of depressive symptoms. The presence of a high FMI increased the likelihood of depressive symptoms, whereas the presence of a high LMI decreased the likelihood of depressive symptoms. Since neither of these variables can exist independently of each other it is important to remember that their opposite effects will moderate each other. This further emphasizes the importance of considering these factors both individually and simultaneously.

A variety of possible explanations for the body composition–depression relationship exists. Research suggests that there may be some genetic overlap between obesity and depressive symptoms, that could be driving both states^43^. Further it has been suggested that the psychological aspects of beauty standards may play a partial role in mediating the relationship between overweight and depressive symptomatology^1^. However, a study with a 12-month follow-up found that increases in fat mass or BMI were not related to any increase in depression^44^.

The most common of the explanations for the body composition–depression relationship is that of an underlying inflammatory effect. Various inflammatory markers, including CRP, have been used as measurements of inflammatory responses within the body^1^, and hsCRP was used in the current study as well. The current study found that high FMI was associated with a higher hsCRP concentration. Considering the association found between FMI and non-melancholic symptoms, it is not surprising that we found higher hsCRP concentration in the non-melancholic group than in the non-depressed group. This is in line with prior research showing that non-melancholic depressive symptoms correlate with CRP levels^12^, and that those having both obesity and metabolic syndrome have the highest levels^2^.

The pathologies of both obesity and depression have inflammatory components^1,15^, with obesity exhibiting chronic low-grade inflammation^1^. Visceral adipose tissue has a particularly high production of pro-inflammatory cytokines that play a role in both obesity and depression^14^. This link between the two pathologies via immunological and inflammatory pathways has been suggested to be bidirectional and self-perpetuating, leading to a vicious cycle of the body composition–depression relationship being exaggerated over time^1^. Research has also been able to show that brain-derived neurotrophic factor, which is associated with obesity in humans, can be downregulated as a result of inflammation driven emotional changes in animals^1^. This provides a possible explanation of how the inflammatory pathway may work, but overall little is still known about the details of the inflammatory connection.

We did not detect any difference in hsCRP concentrations between the melancholic and non-melancholic groups. This may simply be due to the low power of the melancholic group. Melancholic depression has been shown to have lower inflammatory markers than non-melancholic depression^12^, and may hence not be part of this obesity–depression relationship. The current thinking is that melancholic depression is not associated with inflammation, but rather with a change in the HPA-axis regulation^12^. If the thought that inflammation is what causes the changes in body composition is true, then this would explain why there is no association between melancholic depression and higher FMI. To accurately assess changes in the HPA-axis, multiple parameters need to be tested^2^, making any such analysis extremely difficult. It has however been reported that cortisol levels are positively associated with melancholic depression^12^. Further, research involving Cushing’s syndrome has been able to show a causal relationship between cortisol and depression^1^.

It is of importance to note that some participants in the present study had comorbid diseases that could possibly have influenced our findings. Of note are especially cardiovascular disease, which was more prevalent in the high FMI group. Our findings indicate that group B (high fat mass and high lean mass) had the highest prevalence of cardiovascular disease, which is in agreement with previous research indicating that the combination of high FMI and high LMI is in fact predictive of development of diabetes^45^. This same body composition profile was also associated with the highest cardiometabolic risk^45^.

Both lower LMI and lower FMI show a relationship with lower fasting plasma glucose levels. However, glucose metabolism seems to be more related to FMI as indicated by higher glucose concentrations at 30 and 120 min after an OGTT. It has previously been shown that non-melancholic depression is more closely related to higher fasting glucose concentrations than melancholic depression is^12,23^. Here, however, we found no differences between the depressive subtypes for fasting glucose, but at 2 h after the OGTT the non-melancholic group showed higher glucose concentrations than the non-depressed group. Plasma glucose 2 h after an OGTT has been shown to be a better predictor of mortality than fasting glucose^46^. Furthermore, impaired glucose metabolism is known to be a cardiovascular risk factor^46,47^. Since all diabetics were excluded from our study sample the glucose concentrations are within the normal range even though there is a difference between the groups. The values themselves do not infer a greater risk of cardiovascular disease than the general population, but the difference between the group can serve as an indication of some underlying difference in glucose regulation in the non-melancholic group. This is in line with the findings that non-melancholic depression is associated with body composition, as glucose metabolism is often altered in obesity, and both are associated with depression^1^.

In accordance with prior research^33^ we showed that blood pressure has a main effect, with both higher LMI and FMI being related to higher blood pressure. This is true for both systolic and diastolic pressures. For the depressive subtypes we were able to show that the melancholic group has both lower systolic and diastolic blood pressure than either the non-depressed or the non-melancholic group. Higher blood pressure has been associated with both obesity and elevated glucose concentrations in depressed individuals^10^. Melancholic depressed individuals have previously been shown to have lower systolic blood pressure than non-depressed individuals^12^. This is all in line with our findings of the non-melancholic group having a stronger relationship with dysfunctional glucose metabolism and body composition.

Previous studies have reported sex-differences in the body composition–depression relationship^15^, with women displaying a stronger relationship than men^1,17,18^. Obesity and depressive symptoms have been shown to be more closely related for females^48,49^. However, it has been suggested that the difference may be due to the sex-specific body compositions^14^. Others have suggested the presence of a psychological difference in depression between the sexes due to sociocultural factors, as well as some possible differences due to sex-hormones^50^. However, all participating women in this study were postmenopausal. Sociocultural factors, such as perceived beauty standards, have been found to affect females more than males, causing females to have a higher risk of dissatisfaction with their body-type leading to depression^50^. Decreases in estrogen expression can also, through reduced serotonin levels, trigger depression^50^.

It has been found that women report more depressive symptoms associated with anxiety and eating, while men tend experience more symptoms related to substance abuse^51^.

This suggests an interesting possible direction for further studies to look at sex differences and connections to depressive subtypes through lean and fat mass. Ideally all factors in this study could have been analyzed separately for both sexes, which would have allowed us to evaluate for differences between men and women. While we were not able to stratify the analyses according to gender due to limited power in the analyses, we standardized the body compositions factors by sex around their sex-specific means in order to effectively eliminate at least the sex-specific differences in body composition due to the differences in fat and lean mass distributions in men and women.

The current study has both strengths and limitations. Among the strengths are the extensively phenotyped participants and random selection of participants from the pool. The cross-sectional nature of the study is a limitation because it allows for no inferences regarding directionality or time-dependent causality. Other limitations are the presence of comorbidities among participants, and self-reported depressive symptoms rather than clinically diagnosed depressive disorders. The limited subgroup size prevented us from analyzing men and women separately, which would have been of benefit in gaining the most information possible. The fact that fewer male participated in the study is a limitation. Ideally, we would also have had cortisol measurements for all the participants, but that information was not available. The age range could be viewed as a limitation in how these findings can be generalized to the general population, or as a strength since this minimizes the effect of aging on the results.

It would be of interest for further studies to build on these findings by introducing time as a factor. Some previous research has suggested that the body composition–depression relationship could be reinforced over time^15^. Depression has also been shown to have a relationship with long term body composition in some adolescents^16^. Another study showed that during a 12-month follow up increased BMI, visceral adiposity, or body fat did not correlate with increased depression^44^. Some studies have shown that there is in fact a bidirectional relationship between elevated weight and depression for adults^52,53^. Overweight and obese adults have a higher likelihood of developing depression^50^, and depression is predictive of obesity^53^. This has been suggested to possibly be caused by inflammatory markers, hormonal changes, HPA-axis dysregulation, oxidative stress, or psychosocial mechanisms such as negative self-perception and stigmas^50,52,53^. Obese people have a higher likelihood of developing depression than overweight individuals, which suggests the possible existence of a dose–response factor in this relationship^50,53^. Focusing on these same factors as the current study but in relation to lifetime body composition could be interesting. As we know birthweight and changes in weight over a lifetime can affect comorbidities differently^26,27,54^. It may be of value to know if rather than just body composition at a certain time, changes in body composition over the life course are associated with prevalence of one or the other type of depressive symptoms.

Depression is a treatable disease for which there are many effective treatment options^55^. One challenge is the heterogenous nature of depression, and the fact that currently the subtypes are only distinguished based on self-reported criteria rather than established biomarkers^55^. While depression has been increasing in prevalence, up to half of depressed individuals may be inadequately treated^55^, which tells us about the need for a better understanding of this disease. Underlying pathophysiology of the depressive subtypes may be a factor in how depression could be treated more efficiently.

The novelty of the current study is that it provides more specific information differentiating between non-melancholic depressive symptoms and melancholic depressive symptoms and their relationships with FMI and LMI than previously available publications.

## Conclusion

Non-melancholic depressive symptoms are associated with high fat mass and dysfunctional glucose metabolism.

## Supplementary Information

Supplementary Information.

## Data Availability

The data is available from the corresponding author upon reasonable request.
