# Supplementary material for: Non-melancholic depressive symptoms are associated with above average fat mass index in the Helsinki birth cohort study
Source: Sci Rep. 2022 Apr 28;12:6987. doi: 10.1038/s41598-022-10592-3 (PMC9051115; doi:10.1038/s41598-022-10592-3)
Supplement: Supplementary file 1 — Supplementary Information. [file 41598_2022_10592_MOESM1_ESM.docx]

**Supplementary Material**

|  | Excluded subjects  N=182 | Participants N=1510 | P-value |
| --- | --- | --- | --- |
| Women, n (%) | 103 (57) | 842 (56) | 0.83 |
| Age (years), mean (SD) | 62 (3) | 61 (3) | 0.004 |
| Education (years), mean (SD) | 11.8 (3.8) | 12.4 (3.7) | 0.047 |
| Cohabitating, n (%) | 137 (75) | 1142 (76) | 0.88 |
| Current smoker, n (%) | 47 (26) | 356 (24) | 0.50 |
| LTPA (METh/week), mean (SD) | 35.1 (24.6) | 38.5 (27.3) | 0.10 |
| BMI (kg/m^2^), mean (SD) | 27.5 (4.7) | 27.0 (4.2) | 0.16 |

**Appendix 1. Comparison of demographic and lifestyle factors between subjects excluded due to missing data and the participants of the study.**

*Note.* BMI= Body Mass Index, LTPA=Leisure-time physical activity, METh=Metabolic equivalent hours
